# Supplementary material for: Causal Relationship between Aging and Anorexia Nervosa: A White-Matter-Microstructure-Mediated Mendelian Randomization Analysis
Source: Biomedicines. 2024 Aug 16;12(8):1874. doi: 10.3390/biomedicines12081874 (PMC11351342; doi:10.3390/biomedicines12081874)
Supplement: Supplementary file 1 [file biomedicines-12-01874-s001.zip › Supplementary Figure/Figure S8/Leaveoneout plot of MR analysis for TL to ubm-b-2104.pdf]

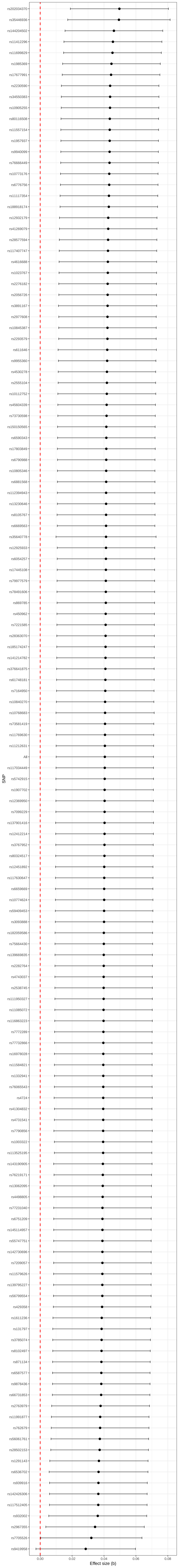

SNP
Effect size (b)
0.00
0.02
0.04
0.06
0.08
rs202034370
rs35446936
rs144204502
rs11412296
rs11699829
rs17677991
rs1985369
rs2230590
rs34550383
rs10905255
rs80116508
rs11557154
rs1957937
rs9940099
rs76666449
rs10773176
rs6776756
rs11117354
rs188918174
rs12932179
rs41269079
rs28577594
rs117407747
rs4616688
rs1023767
rs2276182
rs2056726
rs3891167
rs2977608
rs10845387
rs2293579
rs611646
rs9955360
rs4530278
rs2555104
rs10112752
rs45604339
rs73730598
rs150150565
rs6590343
rs17803849
rs6790988
rs10805346
rs6881568
rs112394943
rs13230646
rs8105767
rs6669563
rs35640778
rs12925933
rs6054257
rs17445108
rs79977579
rs78491606
rs869785
rs450962
rs7221585
rs28363070
rs185174247
rs141214782
rs376641875
rs61746181
rs7164950
rs10840270
rs10768683
rs73581419
rs11769630
rs11212631
A8
rs117034449
rs5742915
rs1907702
rs12369950
rs7099229
rs137901416
rs12412214
rs3767952
rs80324517
rs12451892
rs117630647
rs6659669
rs10774624
rs59409453
rs3093888
rs182059586
rs75664430
rs139669835
rs2282764
rs4743037
rs2538745
rs111950327
rs11085072
rs116863223
rs7772289
rs77732866
rs16978028
rs11584821
rs1332941
rs76065543
rs4724
rs41304832
rs4731541
rs7790856
rs1003322
rs113525195
rs143190905
rs76219171
rs13062095
rs4498805
rs77231040
rs6751209
rs145114957
rs55747751
rs142730696
rs7209057
rs11579626
rs139795227
rs56799554
rs429358
rs1611236
rs131797
rs3785074
rs8102497
rs871134
rs6587577
rs9878436
rs66731853
rs2763979
rs11991877
rs762679
rs56061761
rs28502153
rs1291143
rs6536702
rs939916
rs142426306
rs117512405
rs932002
rs2967355
rs7705526
rs9419958
